# Networking in metabolism and human disease

**DOI:** 10.18632/oncotarget.4561

**Published:** 2015-06-22

**Authors:** Leif Väremo, Jens Nielsen

**Affiliations:** Department of Biology and Biological Engineering, Chalmers University of Technology, Gothenburg, Sweden

A nexus of many complex human diseases and conditions, including type 2 diabetes (T2D), obesity, and cancer, is an altered cellular metabolism. Deviations in metabolism from a healthy phenotype often influence the metabolic network on a global level, rather than exclusively affecting specific pathways. The apparent complexity makes it challenging to study these diseases and require a combination of genome-wide data and innovative holistic analysis approaches [1].

Metabolites are connected to each other through the chemical reaction network and the reactions are connected to their corresponding enzymes, providing a bridge between metabolism and the genome. This structure allows for constructing computer models of metabolism, enabling study of human disease and metabolism at the global level. These models are termed genome-scale metabolic models (GEMs) and can be used for high throughput simulation, contextual data analysis and interpretation, as well as network based analysis and comparison, and are fundamental for the study of metabolism in the area of systems biology [2]. Besides being comprehensive network representations of metabolism, GEMs also contain the stoichiometric information about each reaction so that the system is mass-balanced. Several methods have been developed for using GEMs to simulate and quantify reaction fluxes under different conditions. Furthermore, the inherent metabolite-reaction-gene topology makes these models optimal for integrative analysis of gene expression data, in the context of transcriptional regulation of metabolism [3, 4].

T2D, as a complex metabolic disease, has been studied using GEMs [5]. A central feature of T2D is the development of insulin resistance in several tissues, including liver, adipose and skeletal muscle, thus leading to high glucose levels in the blood. Muscle in particular is important in this context since it is the major site for glucose disposal. Insights into the transcriptional and metabolic changes in diabetic skeletal myocytes are thus important in order to fully understand the pathology of T2D. However, until now there was no available comprehensive myocyte GEM to allow for analysis and contextualization of diabetic muscle transcription data. In a recent study, published in *Cell Reports*, we therefore set out to reconstruct the skeletal myocyte GEM [6]. By generating and integrating genome wide expression data at both the transcript and protein level we were able to determine, for each enzyme, if it is present or absent in skeletal myocytes and thus infer the presence of each corresponding metabolic reaction. This information could then be translated into a GEM, representing the metabolic capability of myocytes, covering 5590 reactions, 2396 metabolites and 2419 genes.

With the aim to characterize the metabolic effects of T2D on skeletal muscle, we connected the results from multiple studies by performing a meta-analysis of six published datasets on T2D muscle gene expression. By integrating all of these condensed data with the myocyte GEM, a metabolic subnetwork emerged that was significantly affected by transcriptional regulation. Using the genes underlying this metabolic signature of T2D, we were able to predict the disease state of individual samples from each separate study, confirming the impact of these genes. In particular, the signature included down-regulation of genes associated with pyruvate oxidation, tetrahydrofolate (THF) metabolism and branched-chain amino acid (BCAA) catabolism.

The patterns for BCAA catabolism and pyruvate oxidation is in line with previous results, but little has been reported about THF metabolism in connection to T2D. Our observation of down-regulated THF metabolism coincided with the results from a pathway analysis, showing transcriptional down-regulation of methionine and nucleotide metabolism, both parts of the metabolism involving THF derivatives. Interestingly, in contrast, the gene *FTCD* was up-regulated pointing to a flow from histidine catabolism to THF metabolism. Histidine has been shown to have positive effects on T2D [7] and it is intriguing to speculate whether increased histidine catabolism in myocytes is associated with the negative effects seen in T2D.

The amount of available data and information in life science is growing and it is essential to exploit and connect data from different sources in order to be able to unravel the biology behind complex diseases. This includes connecting genome-wide data from multiple levels (e.g. proteomics and transcriptomics), connecting analysis results with available gene-level annotation and information (e.g. provided through high-quality GEMs), and connecting and consolidating data from multiple studies. Furthermore, GEMs have the ability to contextualize big data, which often can be hypothesis generating. This is a natural part of systems biology where high-throughput large-scale analyses can pinpoint likely targets of interest, worthwhile to study in more depth. It is therefore also necessary to connect the output from systems biology research with research in molecular biology. With good experimental design, proper data, and analysis approaches that can connect multiple sources of information, successful studies can result in increased mechanistic and molecular understanding of complex diseases, discrimination between causes and effects, and identification of potential biomarkers and novel drug targets.
